# Editorial: Case reports in renal cell carcinoma

**DOI:** 10.3389/fonc.2023.1165013

**Published:** 2023-03-01

**Authors:** Tony Z. Zhuang, Seema M. Mustafa, Kathryn E. Beckermann, Mehmet Asim Bilen

**Affiliations:** ^1^ Department of Medicine, Emory University School of Medicine, Atlanta, GA, United States; ^2^ Department of Medicine, Vanderbilt University Medical Center, Nashville, TN, United States; ^3^ Department of Hematology and Medical Oncology, Emory University School of Medicine, Atlanta, GA, United States

**Keywords:** renal cell carcinoma, biomarker, immunotherapy, next-generation sequencing, cytoreduction

## Background

Renal cell carcinoma (RCC) is one of the most common malignancies worldwide with nearly 81,000 new cases and 15,000 deaths annually (1). Clear cell RCC (ccRCC) is the most common histologic subtype. Immune checkpoint inhibitors (ICI) have been approved as a frontline treatment in recent years, with nivolumab/cabozantinib, pembrolizumab/levantinib, and pembrolizumab/axitinib approved for all risk groups, while nivolumab/ipilimumab is reserved for intermediate-to-poor risk disease (2–7). While the presentation of case reports and series may not represent practice-changing data, we believe it can spark new thoughts regarding biology, mechanisms, therapeutic options, and responses. Despite significant advances in immunotherapy and the discovery of novel biomarkers, the management of RCC is complex, owing to its heterogeneity, with multiple histological and genomic subtypes. This brief review describes 12 unique cases presented by 87 authors exploring the role of next-generation sequencing in treatment selection, rare disease and treatment complications, and the management of oligometastatic disease.

## The role of next-generation sequencing in management of RCC

Mikhaylenko et al. and Huang et al. present cases of papillary RCC. The first patient developed multiple, bilateral type 1 papillary RCC tumors with a germline heterozygous missense variant in *MET*, which promotes unchecked cellular division in hereditary papillary renal cell carcinoma (Mikhaylenko et al.). They discuss oncogenic drivers in SWI chromatin complex disruption and explore the importance of next-generation sequencing for the development of an adjuvant treatment selection. The other patient was found to have an incidental large type 2 papillary carcinoma measuring 15 centimeters, underwent surgical resection, and recovered well without recurrence (Huang et al.). Given that PRCC is a complex tumor with varying genetic and molecular heterogeneity, the selection of targeted therapy with NGS for relapsed/refractory cases may prove to be beneficial.

Lian et al. describe PARP inhibition in a patient with BAP1 mutation as a potential treatment in ccRCC, progressing after TKI/mTOR therapy and intolerance to ICI therapy. The patient originally achieved partial response with a 28-month sequenced regimen of TKI-TKI-mTOR and cytoreductive nephrectomy but later developed CNS and pulmonary metastasis. Next-generation sequencing revealed a frame shift pathogenic mutation in BAP1 (3p21.1) in ccRCC, and niraparib was subsequently started, achieving a partial response of five months. BAP1 mutations across cancer types were identified in RCC, pleural mesotheliomas, cholangiocarcinoma, and ocular melanoma. BAP1 acts as a tumor suppressor and inhibits E3 ligase activity in BRCA1/BARD1. This process inhibits ubiquitination and promotes deubiquitination of existing ubiquinated chains. Wang et al. present a patient with asymptomatic Birt–Hogg–Dube syndrome and a history of spontaneous pneumothorax. She was found to have a concurrent germline and novel somatic mutation in the folliculin gene (FLCN). The authors posit the clinical relevance of a somatic FLCN mutation in conferring familial risk by conducting correlative studies on FLCN, TFEB/TFE3, mTOR, and cilia length. TFEB was found to be constitutively expressed in the nucleus of the FLCN germline-mutated tissue, whereas TFE3, phosphorylated mTOR, and cilia were highly expressed in FLCN-deficient tissue. The authors emphasize the potential role of FLCN inactivation on TFEB/TFE3 migration in the tumor microenvironment. These case reports highlight the growing role of molecular studies in understanding ccRCC disease progression.

Martini et al. and Miroński et al. describe a rare diagnosis of translocation-associated RCC (tRCC), typically seen in the pediatric and younger adult population. Mutations in TFE3 and TFEB define this aggressive, rare RCC variant that lacks targeted treatments. One patient achieved an exceptional response to nivolumab and ipilimumab (Martini et al.). The authors then highlight intratumoral niches rich in TFC1+ CD8+ T cells and lymphovascular invasion as potential biomarkers of response. In another rare case of this, a patient with metastatic RCC underwent left radical nephrectomy and adrenalectomy with adjuvant temsirolimus, resulting in 10-month DFS until palliative radiation was required for spinal decompression (Miroński et al.). These cases highlight poor outcomes in this aggressive variant, with a recent retrospective study reporting mOS of 17.8mo in dual ICI and VEGF treatment (8).

## Rare cases of RCC complications and treatment events

Recent case reports have emerged that describe rare and atypical presentations of RCC. Nkengurutse et al. describe an atypical case of metastatic RCC to the left atrium without IVC involvement complicated by coronary sinus invasion. Surgical resection and coronary sinus repair allowed for complete tumor removal, with the patient recovering well. Yang et al. present a rare diagnosis of anti-N-methyl-D-aspartate (NMDA) encephalitis as a paraneoplastic manifestation of ccRCC. The patient’s symptoms resolved with pulse steroids and IVIG. Billon et al. highlight the development of vitiligo in the setting of a durable complete response with immunotherapy. The patient had progressed on frontline sunitinib for metastatic RCC and achieved a pCR to nivolumab after six months of treatment that was complicated by hyperthyroidism and vitiligo. The authors discuss the rarity of vitiligo as an irAE, which has been associated with a positive response marker, and the unanswered role of immunotherapy discontinuation in pathologic complete response.

Schmeusser et al. describe a complex case in balancing management of solitary kidney tumors and renal function preservation. The patient had a history of childhood Wilm’s tumor treated with right nephrectomy and adjuvant chemotherapy, but they later developed a new malignant left solitary renal mass. A core needle biopsy was unable to differentiate between benign oncocytoma and chromophobe RCC. A complex open partial nephrectomy was performed for tissue sampling and the symptoms of mass effect. The renal function was preserved with perioperative nephrology guidance.

## Management of oligometastatic RCC

Zhuang et al. and Qin et al. report cases of oligometastatic involvement in RCC. The first patient is a Jehovah’s witness who responded to two cycles of nivolumab and ipilimumab and subsequently underwent a successful cytoreductive nephrectomy and a left pulmonary metastatectomy with minimal intraoperative blood loss (Zhuang et al.). The patient continues to demonstrate a complete durable response. The authors then discuss the CARMENA and SURTIME trials and explore the role of secondary cytoreductive nephrectomy in the immunotherapy era. The other patient developed a rare muscle metastasis in RCC involving the masseter (Qin et al.). The patient was able to be observed until the mass effect required surgical resection. The surgical resection was successful, with negative surgical margins and no evidence of disease at follow-up.

In summary, this Research Topic provides a selection of cases highlighting the complexity of RCC diagnosis and treatment in relation to surgical management, rare disease, treatment complications, and the emerging role of next-generation sequencing in the immunotherapy era. We thank the authors for their contributions to this Research Topic.

## Author contributions

TZ and SM drafted the editorial. MB and KB edited the manuscript. All authors contributed to this work and gave approval to the final version.
